# “Diagnosis by Behavioral Observation” Home-Videosomnography – A Rigorous Ethnographic Approach to Sleep of Children with Neurodevelopmental Conditions

**DOI:** 10.3389/fpsyt.2015.00039

**Published:** 2015-03-17

**Authors:** Osman S. Ipsiroglu, Yi-Hsuan Amy Hung, Forson Chan, Michelle L. Ross, Dorothee Veer, Sonja Soo, Gloria Ho, Mai Berger, Graham McAllister, Heinrich Garn, Gerhard Kloesch, Adriano Vilela Barbosa, Sylvia Stockler, William McKellin, Eric Vatikiotis-Bateson

**Affiliations:** ^1^Sleep/Wake Behavior Clinic and Research Laboratory, Department of Pediatrics, Faculty of Medicine, University of British Columbia, Vancouver, BC, Canada; ^2^Faculty of Science, Thompson Rivers University, Kamloops, BC, Canada; ^3^Treatable Intellectual Disability Endeavour, Vancouver, BC, Canada; ^4^Austrian Institute of Technology, Vienna, Austria; ^5^Technical University of Vienna, Vienna, Austria; ^6^Department of Neurology, Medical University of Vienna, Vienna, Austria; ^7^Department of Electronics, Federal University of Minas Gerais, Belo Horizonte, Brazil; ^8^Division of Biochemical Diseases, Department of Pediatrics, Faculty of Medicine, University of British Columbia, Vancouver, BC, Canada; ^9^Department of Anthropology, Faculty of Arts, University of British Columbia, Vancouver, BC, Canada; ^10^Department of Linguistics, University of British Columbia, Vancouver, BC, Canada

**Keywords:** sleep, pediatric, behavioral observation, videosomnography, ethnography, Willis Ekbom disease, restless legs syndrome, neurodevelopmental conditions

## Abstract

**Introduction:** Advanced video technology is available for sleep-laboratories. However, low-cost equipment for screening in the home setting has not been identified and tested, nor has a methodology for analysis of video recordings been suggested.

**Methods:** We investigated different combinations of hardware/software for home-videosomnography (HVS) and established a process for qualitative and quantitative analysis of HVS-recordings. A case vignette (HVS analysis for a 5.5-year-old girl with major insomnia and several co-morbidities) demonstrates how methodological considerations were addressed and how HVS added value to clinical assessment.

**Results:** We suggest an “ideal set of hardware/software” that is reliable, affordable (∼$500) and portable (=2.8 kg) to conduct non-invasive HVS, which allows time-lapse analyses. The equipment consists of a net-book, a camera with infrared optics, and a video capture device. (1) We present an HVS-analysis protocol consisting of three steps of analysis at varying replay speeds: (a) basic overview and classification at 16× normal speed; (b) second viewing and detailed descriptions at 4–8× normal speed, and (c) viewing, listening, and in-depth descriptions at real-time speed. (2) We also present a custom software program that facilitates video analysis and note-taking (Annotator^©^), and Optical Flow software that automatically quantifies movement for internal quality control of the HVS-recording. The case vignette demonstrates how the HVS-recordings revealed the dimension of insomnia caused by restless legs syndrome, and illustrated the cascade of symptoms, challenging behaviors, and resulting medications.

**Conclusion:** The strategy of using HVS, although requiring validation and reliability testing, opens the floor for a new “observational sleep medicine,” which has been useful in describing discomfort-related behavioral movement patterns in patients with communication difficulties presenting with challenging/disruptive sleep/wake behaviors.

## Introduction

In children and adolescents with neurodevelopmental conditions (NDCs) and chronic care needs, sleep problems (SPs) constitute one of the most common parental complaints to health care providers (1). Although SPs have been reported in up to 80% of children with NDCs (2), their role in chronic disease morbidity is rarely recognized, complicating appropriate diagnosis, and treatment (1–3). A structured and rigorous approach is needed to reduce the significant health economic burden for this population (4).

A major reason for this shortcoming is the multifaceted clinical appearance of SPs in this population; SPs may go unrecognized, especially if the parent/caregiver-reported presentation does not match the well-known diagnostic criteria such as sleep apnea (2, 3). Sleep lab assessments do not reveal the full spectrum of possible causes for a SP. A medical ethnographic approach (5–7) using the observational skills of parents and therapists has previously enabled us to better understand reasons for unspecified insomnia (8, 9) and connect novel observations such as sensory processing abnormalities (10) and restless legs syndrome (RLS) (11), which are particularly missed in standard clinical assessments and treated for their daytime sequelae.

Restless legs syndrome is a frequent neurologic disorder (12), which can significantly impact sleep/wake-behaviors from early infancy (12, 13), mimic attention deficit hyperactivity disorder (ADHD) (11) and aggravate mental health problems (14). Current diagnostic criteria are based on patient-reported urge to move and sensory discomfort/pain, mainly in the legs, which typically occur during rest and at bedtime. For children, who are non-verbal and/or unable to express these symptoms in their own words, the International *Pediatric RLS-Consensus Group* recently has suggested “*diagnosis by behavioral observation*^1^” as part of the diagnostic criteria (11). However, behavioral observations at bedtime or overnight are particularly challenging, as they are not accessible to observation in the typical clinical working hours.

To facilitate “behavioral observation” in situations that are not amenable to clinical settings, we have developed a home-videosomnography (HVS) recording system that allows for observations and data collection in the natural setting where sleep occurs (15). Videography has been used in clinical work, e.g., observing sleep/wake-behaviors of infants, for more than 35 years (16), including time-lapse HVS-recordings made in the home setting (17–21). However, there have been no suggestions for the rigorous analysis of time-lapse and/or other video recordings captured by HVS. Our system operationalizes an ethnographic explorative approach, combining narratives with real-time video recording and allowing observations with analyzes of high-quality motion data.

The goal of this article is to present the methodology of a HVS system, which we have developed to observe bedtime and sleep in individuals with NDCs and undiagnosed SPs. Our considerations of choice of hard and software for generating, reviewing, and analyzing sleep video recordings will help investigators to customize technical equipment for their individual research settings. A case vignette shows the utility of our HVS system in screening for RLS associated SPs.

## Methods and Results

### Research environment

The proposed procedures for generating HVS are based on review and test of available hard- and soft-ware (Adriano Vilela Barbosa, Forson Chan, Yi-Hsuan Amy Hung, Osman S. Ipsiroglu, Gerhard Kloesch, Eric Vatikiotis-Bateson). Viewing and analyzing home sleep video assessments have been developed empirically based on our clinical work (Osman S. Ipsiroglu, Forson Chan, Yi-Hsuan Amy Hung, Dorothee Veer, Michelle L. Ross, Sonja Soo, Gloria Ho, Mai Berger, Graham McAllister, Heinrich Garn, Gerhard Kloesch). Most of the work was accomplished during trans-disciplinary summer courses at the University of British Columbia between the years 2011–2013 (Osman S. Ipsiroglu, Sonja Soo, Adriano Vilela Barbosa, Eric Vatikiotis-Bateson, William McKellin) (22).

### HVS-recording system

#### Hardware

*Precondition*: A HVS-recording system consists of an infrared security camera and a microphone connected to a PC netbook through a USB video capture device responsible for converting the analog video signal output by the camera into a digital signal. *Solution*: We opted for a low-budget patchwork HVS-recording system including (i) infrared security camera [e.g., *Lorex High Resolution Color Indoor/Outdoor Camera (model number: CVC6975HR)*]; (ii) video capture device [e.g., *Diamond One-Touch Video Capture (model number: VC500)*]; (iii) netbooks, minimum netbook requirements are Intel Atom CPU N450 @ 1.66GHz and 1 GB RAM, Windows XP [e.g., *Acer Aspire One (model number: NAV50), Acer Aspire One (model number: ZG5), and Gateway LT2016U (model number: KAV60)*] (23, 24); (iv) Microphone [*Nexxtech Desktop Microphone (model number: 2616447)*]. The overall costs of this equipment are ~500 Canadian Dollars. The equipment weighs ~2.8 kg in total, and can be carried or transported in a 6″ × 11″ × 16″ container. The hard and software requirements and initial test series are presented in Tables 1 and 2, respectively.

**Table 1:** **Hardware option test process**.

Options	Hardware			
	Infrared optics	Cost	Storage	Remote control
Spy cameras	Extremely limited	$50–$300	≤8 h	Complex
Consumer-grade camcorders	Extremely limited	≥$1100	<8 h	Complex
Security camera paired with a Netbook	Variety, supports USB adapters	$340–$500	>8 h	Automatic with internet connection

*This table shows the available features and evaluation metrics of the hardware options for HVS-recordings. Hardware options were evaluated based on the flexibility and ease of use of infrared optics and remote control (25). The table shows a comparison of the available camera options, their costs, and storage time. The results show that the only viable option was to utilize the security camera*.

**Table 2:** **Software option test process**.

Options	Software			
	Time Stamp	Audio	Best frame rate (fps)	Internal quality control (QC) with Optical Flow^©^ analysis
Webcam Monitor^©^	Yes	Some	10	N/A corrupted files; QC not possible
Cam Wizard^©^	Yes	Yes	29.97	Frame rates too low for consistent QC
Virtualdub^©^	Yes	Yes	29.97	QC POSSIBLE

*This table shows the available features and evaluation metrics of the software options for HVS-recordings (25). In case frame rates are too low, Optical Flow^©^ analysis becomes unviable due to stuttering and lagging of video frames. Optical Flow^©^ analysis became an internal quality control tool to detect visible or invisible stuttering and lagging phenomena (26)*.

#### Bit-rate software

*Precondition*: The video capture software has to be capable of recording an HVS in real time without dropping frames. To enable stable recording, the software requires a higher sampling rate than the frame rate of the camera. If the camera’s frame rate is too high for the recording software, the video capture software will down-sample the frame rate. In order to keep the amount of processing manageable, the correct codec choice is important. *Solution*: We created a system that records HVS at a resolution of 720 × 480 pixels (NTSC DV resolution) and a frame rate of 29.97 fps (frames per second).

#### Compression software

*Precondition*: Uncompressed video takes up hundreds of megabits per second of video. Available video compression algorithms (*video codecs*) represent a specific trade-off between computational complexity (bit-rate) and output quality. For our purposes, we needed a video codec that was able to (a) produce good video quality, (b) encode the input video stream in real time without dropping any frames, and (c) provide an output bit-rate such that the resulting video recordings would fit in the netbook’s hard disk. *Solution*: After testing a wide range of video codecs we decided to use the MJPEG codec, which met both our bit-rate requirements and our hardware constraints. Our first option was to use an AVC/H.264 codec (high complexity, high quality, low bit-rate). However, our hardware could not handle the high complexity of this algorithm in real time, resulting in dropped frames. The MJPEG algorithm does not perform any inter-frame encoding and therefore has a much lower computational cost. Although the resulting video file sizes were much larger than those produced by more complex codecs, they were still below the limit. HVS requires three to five 10-h recordings; the disk space available for the video recordings in the netbooks was about 200 GB. This limited the data rate to 4 GB/h (Gigabytes per hour).

### Analysis of HVS-recordings

#### Qualitative analysis

**Figure 1:**
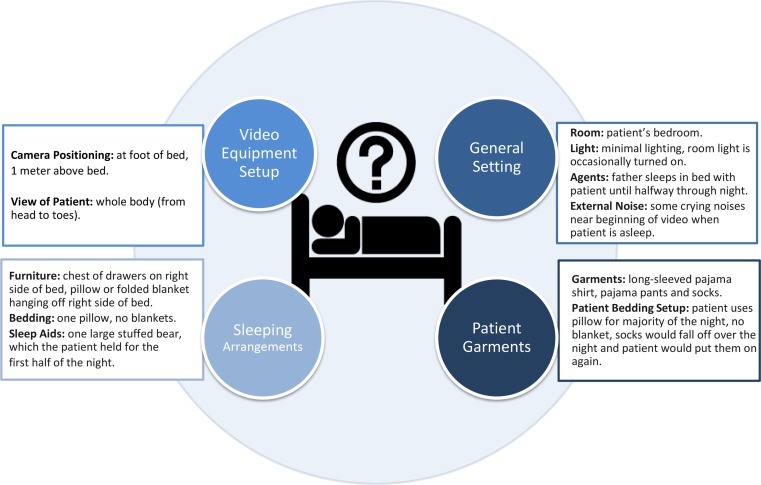
**Setup requirements and domains of qualitative analysis**. This figure gives a graphical overview of the suggested setting for HVS-recordings and the targeted key features, general settings, sleeping arrangements and the patient.

**Figure 2:**
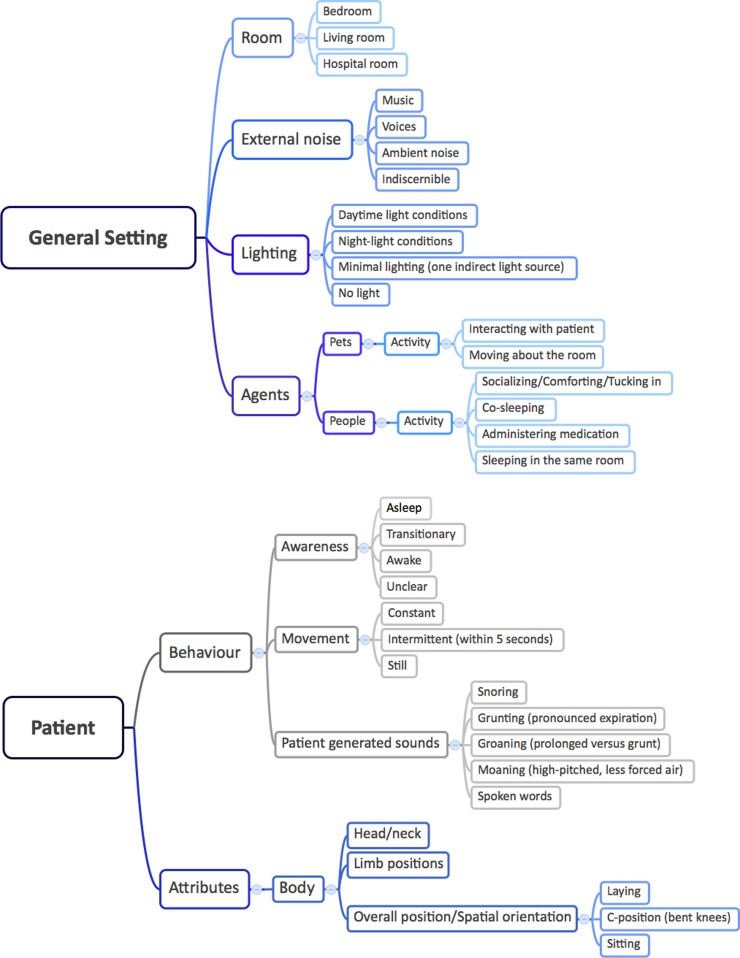
**Mind-maps of the general setting and patient factors**. This figure is a visual representation of the recommended framework for describing the patient’s environment and behavior during the HVS-recording. The patient’s environment is categorized by the external factors that can influence sleep; the patient’s actions are categorized by types of sleep/wake behaviors and sleep positions.

**Figure 3:**
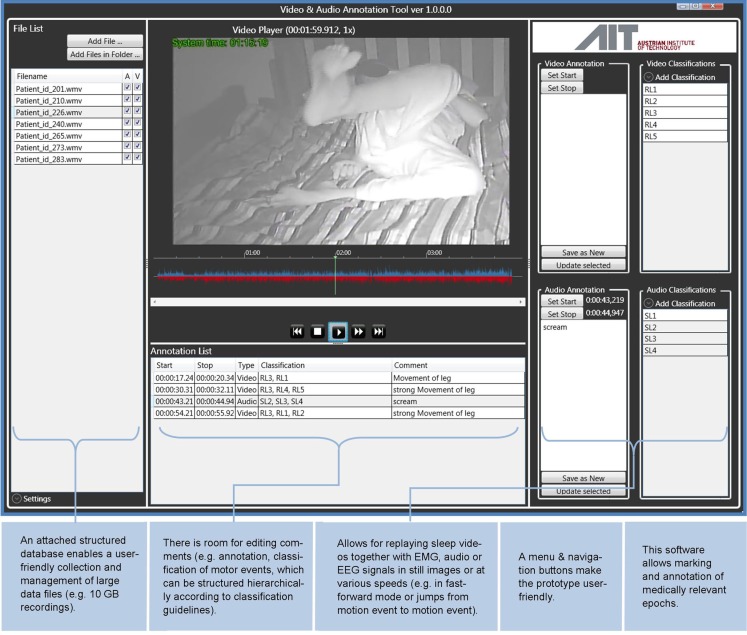
**Annotator^©^ Program**. This figure shows an example screenshot of the Annotator^©^ program (28). Annotation and review of HVS-recordings are carried out in the program (customized software developed by Austrian Institutes of Technology), which allows for viewing at multiple speeds, annotation, marking, and archiving of files in one interface, and eases the process of quantifying descriptive results and generating reports.

*Precondition*: An HVS-recording has to enable: (i) identification of patient behaviors (e.g., movements, patient-generated sound, awareness), attributes (e.g., positioning of the body and different parts of the body), and interactions. (ii) Description of the general setting, including time of the day, sleep environment (e.g., room, bedding situation, lighting, ventilation), and agents (e.g., parents, pets). (iii) The analysis methodology should be considerate of the attention span of the video analyzers, which may change over long periods of viewings, and cause major inconsistencies in the analysis. Figures 1 and 2 provide a graphical overview of the suggested setting aspects. *Solution*: Originally, notes were taken using Microsoft Excel™ with a time stamp. In order to reduce the viewing time and optimize the time-consuming procedure (which also caused reproducibility problems as stopping, writing down and switching between files became competing tasks), we developed, in collaboration with the Austrian Institute of Technology and the Sleep Lab of the Department of Neurology, Medical University of Vienna, customized analysis software, which allows segments of interest to be marked directly. In addition to the marking capabilities of this software, the Annotator^®^ also allows for viewing and re-viewing of HVS-recording in real time and at various higher speeds (2–32 times fast forward), flagging, searching for marked clips, archiving, as well as clipping and archiving video clips in patient files (Figure 3) (27).

#### Quantitative analysis

Based on previous work (29), we decided to use an optical flow algorithm (free downloadable OFA^©^ software) (30) for quantitative movement analysis. OFA^©^ automatically extracts 2D motion from the recorded videos and quantifies frame–frame motion magnitude for the entire video frame and/or selected regions within the frame and stores to disk as time-series data. It further creates a second grayscale video file for visualization of the optical flow field, where pixel velocities are represented by brightness, and motion is computed in both horizontal and vertical directions by comparing every pair of consecutive frames in the video sequence. Regions of interest (RoI) in the scene can be identified, and the overall amount of motion can be computed as a function of time. For quality assurance, mainly detection of stuttering and lagging frames of the video, RoI’s where movements are not expected (e.g., the wall) are reviewed (30).

### Data analysis

The HVS-recording are reviewed at various levels of qualitative and quantitative analysis to ensure comprehensive and efficient data extraction and, later on, visualization of this data. Each level of review follows an individualized yet coherent methodology that is tied together by the main clinical hypothesis. Manual coding of information (i.e., notes or scoring taken during the analysis) can be stored in Annotator^©^ software. The process of reviewing the video, performing qualitative and quantitative analyses, and visualizing the data currently takes ~3–4 h. The use of the Annotator^©^ software for data processing (viewing, annotating, cutting, archiving) has reduced the time commitment by ~30–40%.

### Qualitative data analysis

*Identification of key features*, including the patient behaviors, general setting, and sleeping arrangements; key features set the framework for further analyzes and will be addressed during repeated viewings.

**Figure 4:**
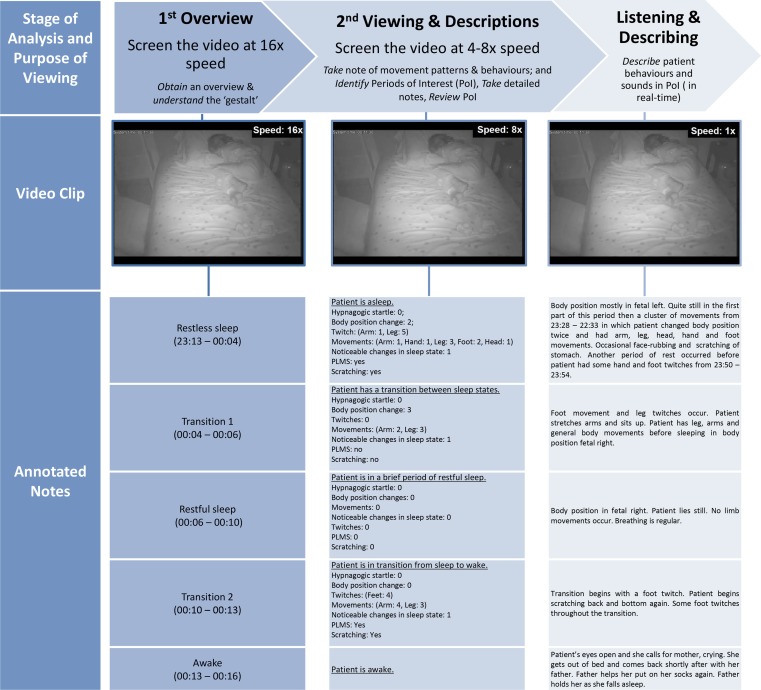
**Steps of analysis**. This figure shows the analysis process for completed HVS-recordings. The annotated notes describe a sample of the case vignette HVS-recording. The video clip, presented at three viewing speeds, corresponds to the “Transition 2” section of the annotated notes.

An excerpt of the key features from the HVS-recording of the case vignette is presented in Figure 4 (time frame 23:13–00:10). (i) Behaviors. In addition to movements and attributes (positioning of the body and different parts of the body), the patient’s interactions with parents, tiredness, and anxious facial expression could be viewed and registered. (ii) General setting and sleeping arrangements: The time is shown digitally on the HVS-recording and allows for orientation; the environment, e.g., room, bedding situation, lightening, and interacting partners, is categorized and presented in Figures 1 and 2.

*Steps of qualitative analysis* obtain information at three levels, (i) *overview*, (ii) *detailed*, and (iii) *in-depth*. The single steps are demonstrated using an excerpt depicted in Figure 4 (18).

(i) Definitions:
  *Sleep:* Eyes closed, stopping movements and actions, usually going along with relaxation of the body;
  *Restless sleep* is defined according to PLM criteria (31): >5 movements of extremities/body within 1 h;
  *Restful sleep*: <5 movements within 1 h;
  *Transition sleep/wake:* not clear whether patient is asleep or awake;
  *Wake:* eyes open, purposeful movements/actions, e.g., communication with parent, which after the HVS study is confirmed by parents for differentiating from parasomnias (e.g., sleep talking).
  Note that after each HVS-recording parents fill out a form for sleep/wake-behaviors with the information about falling asleep time, awakenings, etc.
(ii) First viewing, basic overview and classification: this first overview explores and describes the content of the HVS-recording. The video is played at 16x normal speed from the beginning to end to (a) obtain an overview and identify the key features, which may interfere with sleep; (b) understand the “gestalt” of the behaviors, and (c) identify periods of interest (PoI), which will be the focus of subsequent viewings. Minimal notes inform about the periods of observable qualities of sleep (e.g., restless, restful), awakenings, transitioning periods not easily characterized from the recording, and the behaviors during these waking times. This stage of review helps to develop the main hypothesis (e.g., rhythmic movement disorder or confusional arousals) and thus, subsequent review of the video can be done in an efficient way to support the hypothesis. After fully reviewing the HVS-recording, information about total sleep time (TST), sleep efficiency (SE), and estimates of restful- and restless sleep amounts is recorded. The video summary of the case vignette is presented in Table 3.
(iii) Second viewing and detailed descriptions: the video recording is reviewed more slowly at 4–8× normal speed for more in-depth explorations. At this stage, more *detailed* notes of movement patterns and behaviors are taken, and the *identified* PoIs are verified, i.e., the falling asleep situations, movements, which lead to possible sleep stage changes (arousals). If there are too many PoI, they are categorized depending on the clinical diagnosis (hypothesis), e.g., in RLS, the patterns are analyzed before and immediately after falling asleep, followed by all body-position changes, including how they start and what happens after a body-position change. We use a simplified qualitative analysis of movements derived from the Gross Motor Function Classification System (25). In the case vignette, the *identified* PoIs included the falling asleep situation, the first twenty minutes after falling asleep, 10 min before falling asleep, and around each awakening. Focus was also placed on the patient’s limb movements and body-position changes, as they can fragment sleep.
(iv) Third viewing, listening and in-depth descriptions: the PoIs are *viewed* in real time, in-depth notes are taken and checked for consistency with previous notes. Patient-generated sounds during the PoI are reviewed in real time, and described in-depth. Listening to and reviewing the patient-generated sounds supports discrimination between inspiratory and expiratory snoring. Review of the case vignette HVS-recording through the Annotator software, which visualizes sound digitally and supports discrimination of crying and snoring, revealed missing snoring episodes. This more in-depth level of analysis confirms that the developed clinical hypothesis (e.g., viewing a patient with RLS and fragmentation of sleep) has not changed and the initially identified PoIs were valid for further in-depth analysis.

**Figure 5:**
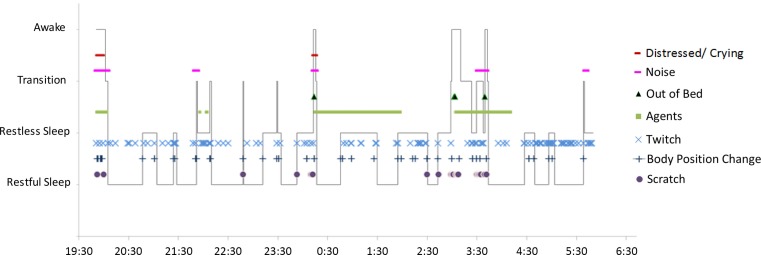
**HVS-hypnogram**. This diagram shows the final hypnogram resulting from analysis and annotation of the case vignette HVS-recording. Time is displayed on the horizontal axis (total period of one night); sleep state (indicated by the solid black line) is displayed on the vertical axis. Sleep events are labeled according to the embedded legend.

*Descriptive data summary* is presented as a semi-quantitative HVS-recording report (Table 3). This summary report includes information about the length of the video (went to bed; lights out; sleep onset; wake time; left the bed; came back, falls asleep). Manual coding information (e.g., notes and/or scoring during analysis) is conducted using the Annotator^©^ software, enable a graphical visualization of the sleep as an HVS-hypnogram (i.e., manual sleep state related coding information on the *y*-axis and time on the *x*-axis; Figure 5).

**Table 3:** **Video summary**.

Video summary	
**Length of video**/Total recording time	**19:51–5:51 (10 h, 0 min)**
**Went to bed**	19:51 (already in bed)
**Lights out**	20:39 (lights fully turned out after already asleep)
**Sleep onset**	**20:05**
Transition from wake to sleep	
**Sleep latency**	**14 min (note that patient was already in bed)**
Total time between going to bed and falling asleep	
**Wake time**	22:48–22:48, 00:13–00:16, 03:00–03:10; 03:40–03:43 (27 min)
Total time in minutes that is scored awake occurring between sleep onset and final wake-up	
**Total sleep period**	**20:05–5:51 (9 h 46 min)**
Period of time measured from sleep onset to final awakening. In addition to total sleep time, it is comprised of the time taken up by arousals and movement time until wake-up	
**Total sleep time (TST)**	**9 h 19 min**
Amount of actual sleep time in a sleep period; equal to total sleep period less movement and awake time	
**Sleep efficiency (SE)**	**93.2%**
Proportion of sleep in the period potentially filled by sleep-ratio of total sleep time to time in bed	
**Restful sleep**	5 h 7 min
**Restless sleep**	4 h 14 min
Persistent or recurrent body movements, arousals, and/or brief awakenings in the course of sleep	

*This table provides the quantitative summary of the case vignette HVS-recording. Video summaries provide information about sleep/wake times, sleep onset, and total sleep period, and allow calculation of sleep efficiency*.

### Quantitative analysis

**Figure 6:**
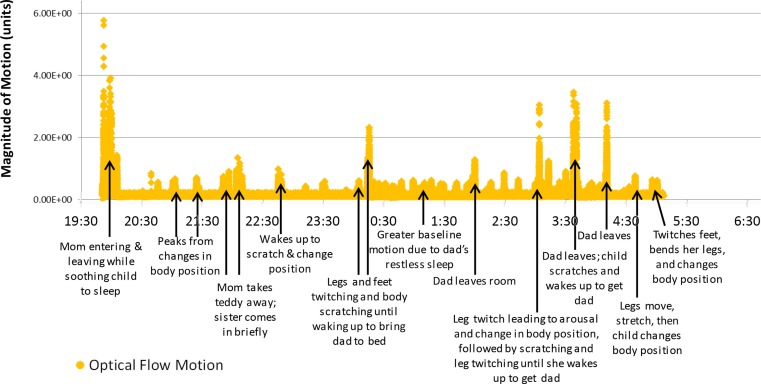
**Optical Flow^©^ diagram**. This diagram shows the results of Optical Flow analysis of the case vignette HVS-recording. Time is displayed on the horizontal axis (total period of one night); magnitude of motion calculated using optical flow (in arbitrary units) is displayed on the vertical axis. Points of interest are labeled to show correspondence between sleep events and peaks of movement intensity.

**Figure 7:**
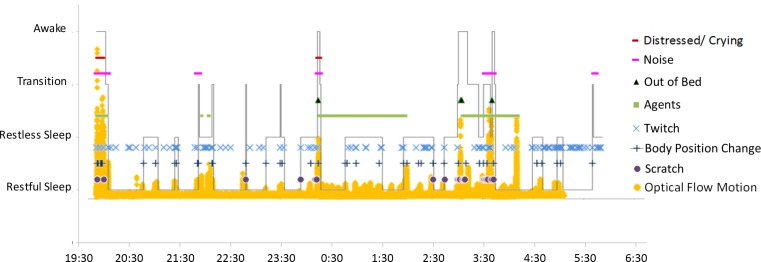
**Comparison between Optical Flow^©^ and HVS-hypnogram**. This diagram overlays the HVS-hypnogram (Figure 5) and the Optical Flow^©^ diagram (Figure 6) to show the close correspondence between sleep state (and number of sleep events) and movement intensity.

The Optical Flow^©^ software/algorithm quantifies frame–frame motion magnitude for the entire video frame and/or selected regions within the frame as time-series data. The software creates a video file where motion amplitude is represented by pixel brightness. When played back, the moving structures (arms, legs, head, etc.) are clearly identifiable and correspond to the derived motion data (29). An Optical Flow^©^ hypnogram (Figure 6) is also generated, which automatically identifies artifacts (e.g., entrance of the father as an artifact) or stuttering phenomena if the speed of the recording is not even. The latter can be used as an internal quality control of the HVS-recording (Figure 7).

### Consent/assent forms

Home-videosomnography has been offered as a research tool with a non-standardized “patchwork” of hard and software. We obtained parental consent for all HVS-recordings. Assent forms are provided when deemed applicable, e.g., patient is age appropriate, usually over the age of 11 years. Both forms are written in lay language that can be fully understood by the target age group and begin with a background explanation of the *purpose* of the home–based video recordings, specifically, that the video study is used to capture the clinical picture of the night time symptoms. Next, the working procedures of the HVS are explained; outlining that the child will be video recorded via an infrared camera during a regular undisturbed night period in his/her area of sleep and that equipment can be couriered without cost to the family. This is followed by an explanation as to why we are asking for consent/assent and, on the consent form, parents and/or caregivers are asked to provide their initials and signatures (signifying their consent); on the assent form, the child is asked to follow the same procedure. Ethics approval by Ethics Review Committee of the Children and Women’s Research Ethics Board, approval #H12-00410-A002.

### Confidentiality/privacy

Home video setup can be facilitated by phone or connection to the Internet; a best practice for keeping confidentiality/privacy has to be worked out. In order to facilitate home setup, netbooks can be remotely accessed from the research lab through an encrypted sync folder. For this purpose, the netbooks have to be connected to the Internet; turning off the Internet connection disconnects and prevents the netbooks from being reviewed by remote access. Parents and/or caregivers are made fully aware of this fact before starting the video study and are able to refuse this part of the service if they prefer. Before proceeding with the HVS, we explained that:

(a) The video system can only be remotely accessed from our research lab;
(b) The video system will be accessed only via the sync folder to help with setup or to check the quality of the HVS-recording;
(c) The connection is encrypted;
(d) We will not access the videos without first notifying the consenting guardian.
(e) Finally, we recommend to the parents and/or caregivers to turn off the netbook’s Internet connection when the remote access service is not being used in order to avoid any breach of confidentiality.

### Location

HVS video recordings are analyzed at the Sleep/Wake-Behavior Clinic and Research Lab at British Columbia Children’s Hospital or Cognitive Sciences Research Labs of the Department of Linguistics, University of British Columbia, to ensure confidentiality of the patient information.

### Overview of patients screened

Since 2010, as a clinical assessment procedure, we have conducted 230 HVS recordings in patients with unexplained/therapy-resistant SPs and NDCs such as ADHD, fetal alcohol spectrum disorders/prenatal substance exposure (FASD), autism spectrum disorder (ASD), global developmental delay and intellectual disability, and chromosomal aberrations (e.g., Down syndrome) (age range: infant to adolescent). The following case vignette shows the utility of HVS in diagnosing RLS in children who are not able to verbally express their complaints and whose daytime sequelae override the clinical recognition patterns of this treatable condition. Table 4, Overview of problems, shows the number of times the HVS equipment was sent to patients’ homes and technical problems encountered with the installation of the various hardware systems in the home setting.

**Table 4:** **Overview of problems**.

Year	Equipment setup	Number of times equipment was sent out	Problems with video equipment		Team
			Type	Count	
2010	Changing software	31	Inconsistent frame rates: “stuttering phenomena” (system/set-up unsuitable for internal quality control)	N/a	One dedicated research assistant, a “technical enthusiast finding solutions”
			No setup problems or damages occurred	N/a	
2011	Webcam^©^	36	Inconsistent frame rates: “stuttering phenomena” (system/set-up unsuitable for internal quality control)	N/a	Several dedicated research assistants, still high dropout rate because of technical difficulties
			Videos repeated due to technical issues, e.g., software crash	12	
2012	Virtualdub^©^	45 (>15 test videos)	No setup problems	43	System/set-up suitable for internal quality control with optical flow; no need for “technical enthusiasts”
2013 – …	Virtualdub^©^	56	Netbook crashes while recording	N/a	System/set-up suitable for internal quality control with optical flow; no need for “technical enthusiasts”
			Video only records for a few minutes	N/a	
			Infrared does not activate	N/a	
			Video records onto netbook but file is corrupt/does not play	N/a	
			Broken equipment	7	

*This table shows the history of recording errors with the HVS system. The main problems with HVS-recordings were caused by software and/or hardware incompatibilities, or accidental changes of netbook default setting by parents during the set-up*.

## Case Vignette

### Past and present history

The female patient was referred to our sleep clinic at the age of 5.5 years for the assessment of intractable behavioral insomnia. Medical history revealed that she was a very active fetus with constant and unusual intensity of intrauterine movements. SPs became a growing concern in early infancy. She could only fall asleep when tightly swaddled. SPs continued to increase over the years including difficulty falling asleep and maintaining sleep.

At the age of 4 years, a sleep lab-based assessment revealed no specific diagnosis, and she was diagnosed with behavioral insomnia and treated with melatonin, in addition to her already diagnosed sensory processing disorder, reactive attachment disorder and ADHD. A treatment trial with two different psycho-stimulants at the age of 5 years resulted in major stress and distress in the family. Parents described their daughter’s behaviors as “drugged down and emotionally labile”; medication was stopped after two weeks when the girl asked her parents not to be forced to take the “crying pills” anymore.

At the time of the assessment, in our clinic, the patient was able to fall asleep within 15–45 min, but she was not able to maintain sleep for more than 1.5 h. She took melatonin at bedtime, which during the course of time had been increased to up to 20 mg without major improvements of her falling asleep or sleep maintenance problems (28). Repeated episodes of awakenings were accompanied by challenging behaviors (e.g., irritable, angry, and anxious). Furthermore, while she was asleep, the patient was described to “thrash” and “kick” around, sometimes hitting both parents, who had decided to co-sleep with her due to her anxiety. Both parents described this situation as very disruptive and they had “bruises all over their bodies” from the patient’s kicking movements and fidgetiness during sleep. When she woke up in the morning she was groggy and moody. Because of her challenging/disruptive behaviors she had been removed from daycare.

### Family history

The patient’s father was described as a “fidgety” person who struggled with sleep disturbances. He would toss and turn at night, talk in his sleep and grind his teeth, and would also wake up “groggy” (indicative of non-restorative sleep). He had been prescribed a non-benzodiazepine hypnotic agent (zopiclone), which did not make a substantial difference. The patient’s paternal aunt had SPs similar to her brother, which started at a young age. The patient’s maternal great-grandfather had been formally diagnosed with restless legs syndrome; the patient’s grandmother and mother both seemed to sleep well. However, the patient’s mother did experience periodic limb movements in wakefulness and, by history, also in sleep.

### Clinical exam

*Clinical exam* was unremarkable. Neurologic exam showed no evidence of focal signs; she was reported to be clumsy and have some developmental coordination disorder. Behaviorally she was alert and under massive tension, listening to the conversation but not interacting with the medical team. Baseline ferritin level was found to be 25 mcg/l (considered low with regards to RLS); complete blood count, including hematology panel, and routine chemistry lab tests were all normal.

### HVS

To further explore her nighttime behaviors we conducted an HVS-recording. Of the three nights recorded, two were analyzed in total and the summary of the first recording is presented in Table 3. In addition, annotations for an excerpt of the HVS-recording, showing the corresponding notes and descriptions for various viewing speeds, are shown in Figure 4. The HVS-hypnogram summarizes the quantified descriptive data (Figure 5), the optical flow hypnogram summarizes the automated quantified analysis (Figure 6), and the comparison between both hypnogram versions presents the high correspondence (Figure 7).

Overall: (i) The patient was able to fall asleep with the support of melatonin and her parents after 14 min; (ii) before falling asleep she showed twitchy leg movements along with a distressed presentation; (iii) the movements also continued after falling asleep and (iv) the patient woke up four times overnight. Each awakening was initiated by a cascade of twitching movements, leading to leg movements and scratching and concluded by the patient getting out of bed to seek the aid of her father; (iv) most striking were the facial expressions of the patient, which showed her desperateness, anxiety, and crying. This nighttime suffering was affecting her daytime behaviors that had been labeled as challenging/disruptive by previous assessments; (v) none of her movements were dystonic, hyperkinetic, or bizarre (which would have suggested nocturnal frontal lobe epilepsy manifestations); (vi) the HVS revealed the periodic limb movements of her mother in the falling asleep situation, and (vii) of her father, each time he came to soothe her; as well, the patient and her father presented with the same stereotypic scratching behaviors.

### Conclusion

In addition to the clinical assessment, the HVS-recording demonstrated the dimension of the clinical RLS diagnosis causing the following cascade: (i) insomnia, difficulty falling asleep and maintaining sleep; (ii) insomnia-related challenging behaviors (increasing anxiety before bedtime resulting in bedtime resistance, and secondary behavioral insomnia), (iii) non-restorative sleep due to periodic limb movements in sleep causing major fragmentation of the patient’s sleep, and (iv) parasomnias (the discussion of the HVS-recording results with the parents helped us to identify hypnagogic hallucinations as an additional cause of anxiety). In addition, the HVS-recording revealed that both parents (including the father who had not attended the clinical assessment) are on the RLS spectrum.

### Course of development

We treated the patient’s RLS-related discomfort with iron supplementation and gabapentin (26) and low-dose melatonin. Overtime, her SPs and challenging/disruptive behaviors diminished; she became able to consistently initiate sleep within 15 min and sleep through the night. She became more aware of her sensory processing abnormalities and participated actively in the titration of the medication with gabapentin. After ~9 months of treatment, her initially delayed biking skills improved to the degree where she could take part in BMX riding events. Her anxiety dissipated. With advancing age, the girl began to articulate her sensations, discomfort and pain in her feet and legs. Retrospectively, it became clear to us that her inability to discriminate between comfort and discomfort was due to a missing “reference” point and inability to articulate the problem, as she had never experienced a comfortable going-to-bed situation and an uninterrupted good night’s sleep. We suggested that after a treatment period of ~3 years, all the patient’s daytime diagnoses would need to be re-evaluated.

### Summary

We report a patient with chronic and early onset familial RLS where, at the initial assessment, the patient herself could not find the words or the means to explain the problem despite being an articulate child. HVS-recordings played a key role in identifying the clinical diagnosis RLS, which responded well to treatment with iron supplements and gabapentin.

## Discussion

For almost 40 years, time-lapse video recordings have inspired physicians; however, technical complexity requiring intensive labor and bulkiness of equipment have prevented their widespread popularity and usage in medical practice (16, 32). In pediatric sleep medicine, videos have been used for research and to exclude artifact-related data during sleep and/or detect respiratory events (19), but despite an increasing interest in videosomnography for REM sleep behavior disorders and epilepsy in adults (33–36), their use for sleep/wake-behavior analyzes and their association with ADHD and/or RLS in children has been limited (37).

Actigraphy has been suggested as the current gold standard of sleep/wake-rhythm assessments (38, 39). Although actigraphy shows more than 80% agreement with overnight polysomnographic laboratory-based studies (38), actigraphy has poor agreement for detecting nocturnal awakenings compared to video observations (38, 39). Actigraphy can miss the clinical significance of major movements such as getting up or jumping on the bed (which are typical movement patterns of children with ASD and RLS) (32, 40). HVS provides all necessary information, and has the advantage that any marked PoI on the hypnogram can be visually re-reviewed and analyzed further.

Cardiorespiratory-video or Level III home PSG’s (41, 42) have been trialed in various settings in typically developing children (43) and in some children with NDCs (44). However, the frequency of artifacts due to the loss of leads has been considered as a significant challenge, not to mention poor tolerability by children with challenging/disruptive behaviors and the challenges parents have to face during the data collection process.

We initially developed HVS to verify caregivers’ narratives about SPs in their child’s natural setting and have been able to improve our understanding of the dimensions of sleep fragmentation through generalized body movements. The case vignette demonstrates the dimension of chronic and early onset RLS-related insomnia, negatively impacting developmental coordination, sensory processing, mental health, causing anxiety, and mimicking ADHD-like behaviors. HVS-recordings not only enabled us to assess the dimension of the falling asleep and sleep maintenance problems but it also gave us insight in the patient’s burden of suffering as demonstrated by her distressed facial expressions, and her help-seeking interactions with her parents. Interestingly, the HVS-recordings also revealed information on the familial nature of the patient’s and her parents’ RLS presentations. Further, HVS prompted us to understand the impact of trivial phenomena, e.g., *scratching*, which is likely not mentioned in most sleep studies. Scratching can be an expression of nocturnal paroxysmal arousals with motor behaviors during sleep (36) or can be associated with RLS-related discomfort as demonstrated, but has never been investigated in a systematic way in the sleep of children.

Complex movements, associated with changes in body position, are proxies for typical neuro-physiological arousals (45), which are detected by full polysomnography including EEG recording. In contrast to neuro-physiological information retained by complex sleep lab equipment, the use of a simple HVS-recording gives basic and limited, but valuable, clinical information. In the context of clinical RLS research, the discrimination of restless and restful sleep at the first viewing level allows for most valuable information about the degree of restorative or non-restorative sleep. Further detailed and/or in-depth analyses of the movement patterns, mainly counting of body-position changes and how they are initiated, presents more valuable information at the next levels of viewing.

Due to its completely non-invasive character, HVS has particular advantages in children with autism or FASDs, who do not tolerate the attachment of any instrumentation on their body due to their sensory processing abnormalities. HVS might also have a value in adult patients (e.g., with dementia or after a stroke), who are unable to express their sensations.

**Figure 8:**
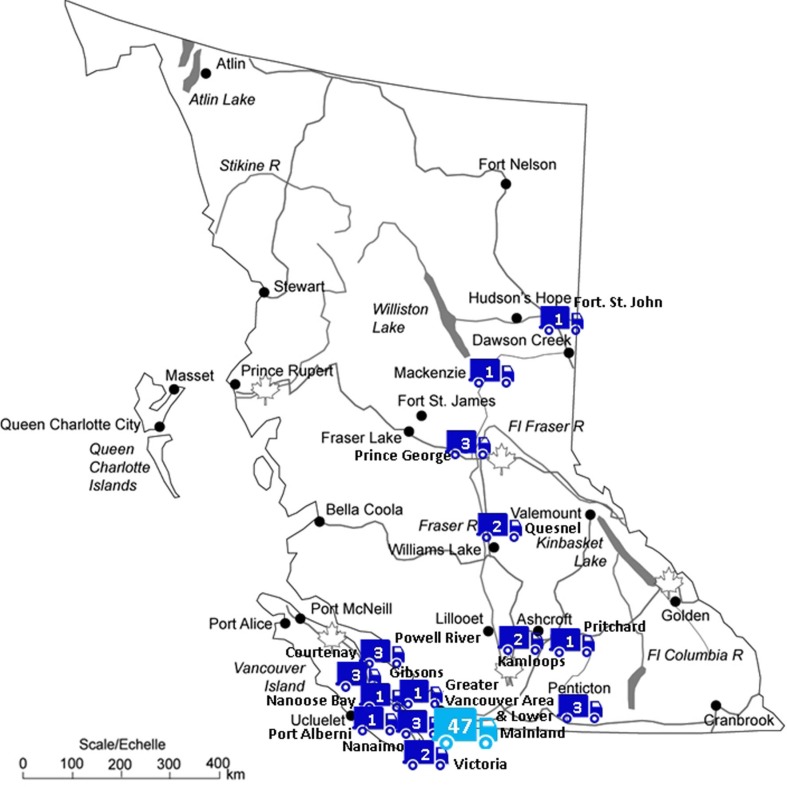
**Map of BC with video equipment destinations over the last period of year**. This figure shows the distribution and frequency of video equipment destinations throughout British Columbia, Canada. There were 74 deliveries in total over the last year, 47 of which were within the *Greater Vancouver Area and Lower Mainland* (distributed by municipality as follows: Coquitlam: 5, Vancouver: 8, Surrey: 9, Burnaby: 6, Abbotsford: 4, New Westminster: 2, Chilliwack: 1, Delta: 3, North Vancouver: 3, Port Coquitlam: 1, Port Moody: 1, South Surrey: 1, Fort Langley: 1, Langley: 1, Maple Ridge: 1). Original map: royalty-free image^©^ Bruce Jones Design Inc., 2009.

Portable and robust hardware and user-friendly video analysis software make our system practical for use in various home settings. We were able to send our equipment across the entire Province of British Columbia, bridging enormous geographical distances (see Figure 8). Our phone and online service to assist parents with installation of the HVS system was used in the initial stages; however, after some adjustments, the need for these services has decreased.

The main problems encountered in our experience have been due to soft and hardware incompatibilities or unintentional changes of netbook default setting. These are problems that can usually be solved by research assistants or sleep technicians through the remote access sync folder without major challenges. Return rate, demolition, and damage of equipment is another factor that has to be taken into account when medical devices are delivered into patients’ homes. As shown in Table 4, in our experience these problems were insignificant. Finally, security and confidentiality of data obtained from home settings are an issue that needs to be tackled carefully. Customized consent forms and hospital-based data security support helps overcome this potential hurdle.

At its current stage, HVS serves as a screening tool providing observational information from situations not amenable to routine clinical sleep assessments. Expansion of its utility as a diagnostic and monitoring tool for sleep disturbances, particularly when associated with abnormal body movements, is feasible. The standardization of the technical settings and of the video analysis procedures described here, will allow for validation studies needed to determine its sensitivity and specificity as well as strengths and weaknesses compared to established methods such as polysomnography and actigraphy.

The customized Annotator^©^ software (27), which in our experience has reduced HVS-analysis time from 4–5 h for a trained individual to 2–3 h, will enable testing of larger numbers of individuals and accomplish further methodological evaluation such as intra- and inter-rater variability and establishment of movement patterns during sleep in a normal, age-stratified population.

In conclusion, while traditional sleep lab studies inform about neuro-physiological, cardio-circulatory, and respiratory surrogate markers, the HVS allows for clinical description of the symptoms associated with a SP, such as going to bed/falling asleep behaviors, nighttime awakenings, sleep-related movements and the associated family interactions. Advanced digital image processing methodologies will enable semi-automatic detection of featured movement patterns and will support automated analysis (30, 46, 47) opening the floor for observational sleep/wake-behavior research in infants, children, youth and adults with movement disorders, neuromuscular conditions and various behavioral conditions. In clinical practice HVS will have its place for phenotyping challenging and disruptive behaviors, which are not easily characterized upon clinical sleep assessments and not captured by traditional lab-based studies. We also expect that HVS will particularly be useful in the recognition of so called “restless” sleep caused by RLS and periodic limb movements as well as for monitoring of interventions.

## Conflict of Interest Statement

The authors declare that the research was conducted in the absence of any commercial or financial relationships that could be construed as a potential conflict of interest.

## Footnotes

^1^Diagnosis by behavioral observation could be particularly helpful for children younger than 6 years of age. In addition, children with neurodevelopmental disabilities that involve language or cognitive impairments could potentially benefit from this approach (14).
